# Supplementary material for: miR-489 Confines Uncontrolled Estrogen Signaling through a Negative Feedback Mechanism and Regulates Tamoxifen Resistance in Breast Cancer
Source: Int J Mol Sci. 2022 Jul 22;23(15):8086. doi: 10.3390/ijms23158086 (PMC9331933; doi:10.3390/ijms23158086)
Supplement: Supplementary file 1 [file ijms-23-08086-s001.zip › ijms-1813608-supplementary.pdf]

Supplementary Data

Supplementary Materials and Methods

Cell Culture

Cells were grown under standard conditions. Cell lines were tested for Mycoplasma via PCR using the Universal Mycoplasma Detection Kit (ATCC). MDA-MB-231, MDA-MB-468, BT549 and Hs578T were cultured in DMEM containing 10% FBS. MCF7-Vec and MCF7-HER2 cells were cultured in DMEM containing 10% FBS with insulin. T47D, HCC1954, BT474 and ZR-75-1 were cultured in RPMI containing 10% FBS. MCF7- WT and MCF7-TAMR cells were cultured in phenol red free low glucose DMEM supplemented with 10% FBS, non-essential amino acids and insulin. For estrogen deprivation studies, MCF7 cells were cultured in phenol red free DMEMF12 supplemented with insulin and 10% charcoal stripped FBS. T47D cells were cultured in phenol red free RPMI and 10% charcoal stripped FBS. All cells were cultured at 37°C in a humidified incubator containing 5% CO2.

Real-time PCR analysis

RNA samples were prepared by using Trizol according to the standard protocol. Real-time RT-PCR was carried out using RT2 Fast SYBR® Green/ROXTM qPCR Master Mix (SABiosciences). Data analysis was performed using the 2– ΔCT method for relative quantification, and all sample values were normalized to the glyceraldehyde-3-phosphate dehydrogenase (GAPDH) expression value (as the internal reference control).

Western blotting analysis

Protein samples were prepared by using M-PER protein extraction buffer (Cat # 78501, Thermo Scientific) added with 10% protease inhibitor, 2% Sodium Orthovanadate (1mM), 1% Sodium Floride (10mM). Immunoblotting was carried out as previously described [1].). All of the antibodies below were purchased from Cell Signaling. p-STAT3 (Tyr705, Cat. #9145); p-p65 (Ser536, Cat. #3033); p-ERK (Thr202/Tyr204, Cat. #4370); p-AKT (Ser473, Cat. #4058); p-IkB (Ser32, Cat. #2859); p-HER2 (Tyr1221/1222, Cat, #2243); STAT3 (Cat. #4904); p65 (Cat. ##8242); ERK (Cat. #4695); AKT (Cat. #4691); IkB (Cat. #4814); HER2 (Cat. #4290); Src (Cat. #2109) and β-actin (Cat. #3700). GAPDH (Cat. #SC-25778) was purchased from Santa Cruz biotechnology.

Cytoplasmic and nuclear fractionation

Cells were seeded in 6-well culture dish, transfected with miRNA or miR-489 mimic or treated with DMSO or p38 MAPK inhibitor SB2350. At 72 hrs post-transfection, cells were harvested and resuspended in 200uL of Hypotonic buffer containing 10mM HEPES (pH 7.9), 1.5mM MgCl2, 10mM KCl, 0.5mM DTT. The cells were homogenized using dounce homogenizer to break open the cells using 10 strokes of a tight pestle. The solution was centrifuged at 1000 rcf for 5 min to precipitate the cell debris and nuclei. The pellet was resuspended with 100uL S1 buffer containing 0.25M sucrose and 10mM MgCl2. 100uL of another solution, S2 containing 0.35M sucrose and 0.5mM MgCl2 was added in another tube. S1 suspended solution was layered over S2 by slowly pipetting S1 over S2. The solutions were centrifuged at 3200 rcf for 10 mins. The supernatant was removed, and the nuclear pellet was resuspended in Lysis buffer for protein estimation and running western blot.

Flow Cytometry

CD24 and CD44 antibodies (BD Biosciences) were used to examine cancer stem cell population. After the treatment cells were harvested and washed with PBS. Cells were then resuspended in 1X PBS+2% FBS solution and counted using 112 Biorad cell counter. 200K cells from each sample were transferred to fresh tube, centrifuged and resuspended in 100uls of 1X PBS+2% FBS. Cells were incubated with CD24-PE and CD44-FITC antibodies for 30min with mixing every 10 minutes. Cells were washed with PBS at the end of incubation, resuspended in 1X PBS+2% FBS and read in BD Accuri C6 flow cytometer.

Immunofluorescence

After indicated treatment, cells were washed with 1X PBS and fixed using 4% paraformaldehyde for 15minutes. Cells were washed thrice with 1X PBS and blocked using 1% Rat Serum, 0.01% triton X-100 and 1X PBS for 1 hour at room temperature. Cells were then incubated overnight with ERα antibody (Santacruz biotechnology) at 4 C. Next day, cells were washed with PBS thrice and incubated with Alexa fluor 594 goat anti-rabbit IgG H&L (Thermo scientific) for 1 hour at room temperature. After 3 washes with PBS, cells were counterstained with DAPI for 15 minutes and mounted on slide. Cells were then imaged using confocal microscope using cy3 and DAPI channel.

Mammosphere Assay.

Cells were trypsinized and mechanically separated and passed through 40-μm filters to obtain single cell suspensions. Cells were plated at 10,000 cells per plate density in super–low-attachment plates in mammosphere media and cultured for 6-8 days. Quantification of mammosphere numbers was accomplished by transferring in 96 well plate.

Estrogen and progesterone treatment

To examine effect of estrogen and progesterone on gene expression and proliferation cells were first cultured in estrogen deprivation condition for 3-6 days. For estrogen deprivation, MCF7 cells were cultured in phenol red free DMEMF12 supplemented with insulin and 10% charcoal stripped FBS. T47D cells were cultured in phenol red free RPMI and 10% charcoal stripped FBS. Cells were then trypsinzed and seeded in desired cell culture dish and treated with estrogen and/or progesterone for indicated amount of time. To examine effect of miR-489 on estrogen dependent proliferation, estrogen deprived cells were treated with control siRNA, miR-489 mimic or miR-489 inhibitor for 6days followed by MTT assay to examine proliferation and colony formation assays to examine effect on progenitor/cancer stem-like cell populaiton.

Microarray analysis

T47D cells were seeded in 6-well culture dish, treated with 28nM scramble miRNA or miR-489 mimic for 72hrs. RNA was extracted with Trizol reagent, followed by clean-up and DNase I treatment with QIAGEN RNeasy mini kit in accordance with the prescribed protocol provided with the kit. Quality control was performed with Agilent Bioanalyser before performing microarray. The data were normalized using the default quantile normalization with R/bioconductor package lumi version 3.2.2. The microarray data in this manuscript is available on the GEO database (GSE99728). A subset of identified genes was validated by q-PCR.

Table S1. List of top dysregulated miRNAs in all three tamoxifen resistant cell lines.

miRNA	MCF7-TAM		MCF7-HER2		MCF:2A	
	logFC [a]	p Value	Ratio (HER2/Vect)[b]	SD	Fold Change [c]	p value
miR-135b-5p	-2.89	1.85E-08	24.6	10.15	4	5.40E-08
miR-20b-5p	0.67	2.36E-03	0.94	0.18	6.5	4.40E-13
miR-218	1.68	5.66E-06	1.41	0.63	6.1	2.20E-14
miR-33b-5p/ 33a-5p	-1.1	1.05E-04	3.4	1.00	2.5	2.80E-04
miR-342-5p	-1.58	3.33E-06	0.73	0.23	−2.1	3.80E-02
miR-378a-3p	0.678	1.46E-03	1.54	0.34	1.6	3.90E-02
miR-489	-3.45	8.23E-07	0.11	0.02	−5.7	1.10E-03
miR-505-3p	-1.27	2.11E-04	0.8	0.09	2	1.30E-02

Note: [a] LogFC, log2 fold-change. Microarray was performed to measure microRNA expression in WT MCF7 and MCF-7-TAM cells. Fold change was calculated simply as the ratio of the microRNA expression level in MCF7-TAM vs. WT cells [2].

[b] RT-PCR analysis was performed to measure microRNA expression. Fold change was determined using the comparative threshold cycle (Ct) method from the ΔΔCT values with the formula $2^{-\Delta\Delta CT}$ and the ratio of microRNA expression in MCF-HER2 vs. MCF7-Vector control cells was presented[3].

[c]The expression of miRNA in MCF7:2A and MCF7 cells was measured using nCounter NanoString assays. The normalized NanoString counts were used to calculate the fold changes of microRNA expression in MCF7:2A vs MCF7 cells[4].

Table S2. The association of miR-489 expression with clinical parameters [a]

	miR-489 Low n(%)	miR-489 High n(%)	Chi-Square P value
**Metastasis**			
Negative	619(47.5)	326 (25.0)	
positive	249 (19.1)	108(8.3)	0.1664
**Node Status**			
Negative	458 (35.1)	248 (19.0)	
Positive	410(31.5)	186 (14.3)	0.1350
**Grade**			
null	42(3.2)	23 (1.8)	
1	57 (4.3)	49 (3.8)	
2	286 (21.9)	216 (16.6)	
3	483 (37.0)	146 (11.2)	< 0.0001
**Pam 50 Subtype**			
Basal	164(12.6)	15 (1.1)	
HER2	89(6.8)	23 (1.8)	
LumA	299 (30.0)	269 (20.7)	
LumB	254 (19.5)	100 (7.7)	
Normal	56 (4.3)	26 (2.0)	< 0.0001
**Old (>55)**			
**≤55 years**	313 (24.0)	124 (9.5)	
**>55 years**	555 (42.6)	310 (23.8)	0.007
**ER Status**			
negative	257 (19.7)	42 (3.2)	
Positive	611 (46.9)	392 (30.1)	< 0.0001
**PR Status**			
negative	458 (35.2)	141 (10.8)	
Positive	410 (31.5)	293(22.5)	< 0.0001
**HER2 Status**			
negative	738 (56.7)	391 (30.0)	
Positive	130 (10.0)	43 (3.3)	0.0111
**Tumor Size**			
≤2cm	361 (27.7)	218 (16.7)	
>2cm	507 (38.9)	216 (16.5)	0.0031

Note: [a] The clinical data was retrieved from the published dataset [5].

Supplementary Figure Legends

Fig. S1. Combination index (CI) analysis of the synergy between miR-489 and tamoxifen. **C**ells were transfected with miR-489 mimic in the presence different concentrations of tamoxifen for 72hours followed by MTT based viability assay. Fraction affected (Fa) versus CI plots were generated using the Compusyn software to determine the extent of synergy between miR-489 and tamoxifen in MCF7-TAM (**A**) and MCF-HER2 (**B**) cells. DRI, dose-reduction index.

Both MCF7-Vec and HER2 are sensitive to miR-489 mimic. A. Colony formation assay. Cells were treated with indicated microRNA mimics or inhibitors for 72 hours followed by colony formation assay for 7-10 days. **B.** Western Blot analysis of ERα signaling pathways.

Fig. S2: Generation and characterization of miR-489 KO cells lines. A. Schematic diagram showing process of miR-489 KO cell line generation. **B.** RT-PCR analysis of miR-489 expression in WT and KO cells. **C.** WT and KO cells were seeded in 96 well plate for 96hrs. MTT based viability assay was performed to determine growth rate. **D.** Hormone starved WT and KO cells were seeded in 12- well plate and treated with ethanol or E2 for 6-days and cell viability was measured by crystal violet staining.

Fig. S3. In silico data analysis of miR-489 expression in ER+ breast cancer patients. A-B. Expression analysis of patient datasets showed inverse correlation between miR-489 signature and estradiol signature (**A**)and miR-489 signature and PI3K-ErBB2 signature (**B**). **C.** Loss of miR-489 expression predicts poor survival in ER+ breast cancer patients.

Fig. S4. The effect of miR-489 and p38 inhibitor on ER localization. A. Immunofluorescence analysis of ERα in miR-489 WT and KO T47D cells. **B.** T47D cells were transfected with

scramble or mimic or treated with DMSO or p38 MAPK inhibitor SB23508 and ERα localization was examined using immunofluorescence.

Fig. S5. In silico data analysis of p38 MAPK signature in ER+ breast cancer patients. A. Analysis of patient dataset showing higher p38 MAPK score in pre-menopausal patients compared to post-menopausal patients. **B**. Analysis of patient dataset showing higher p38 MAPK score in pre-menopausal patients with high grade breast cancer compared to low grade breast cancer.

Supplementary references

1. Lo PK, Kanojia D, Liu X, Singh UP, Berger FG, Wang Q, Chen H: **CD49f and CD61 identify Her2/neu-induced mammary tumor-initiating cells that are potentially derived from luminal progenitors and maintained by the integrin-TGFbeta signaling**. *Oncogene* 2012, **31**(21):2614-2626.
2. Ward A, Balwierz A, Zhang JD, Küblbeck M, Pawitan Y, Hielscher T, Wiemann S, Sahin Ö: **Re-expression of microRNA-375 reverses both tamoxifen resistance and accompanying EMT-like properties in breast cancer**. *Oncogene* 2013, **32**(9):1173-1182.
3. Patel Y, Shah N, Lee JS, Markoutsa E, Jie C, Liu S, Botbyl R, Reisman D, Xu P, Chen H: **A novel double-negative feedback loop between miR-489 and the HER2-SHP2-MAPK signaling axis regulates breast cancer cell proliferation and tumor growth**. *Oncotarget* 2016, **7**(14):18295-18308.
4. Bailey ST, Westerling T, Brown M: **Loss of estrogen-regulated microRNA expression increases HER2 signaling and is prognostic of poor outcome in luminal breast cancer**. *Cancer Res* 2015, **75**(2):436-445.
5. Dvinge H, Git A, Graf S, Salmon-Divon M, Curtis C, Sottoriva A, Zhao Y, Hirst M, Armisen J, Miska EA *et al*: **The shaping and functional consequences of the microRNA landscape in breast cancer**. *Nature* 2013, **497**(7449):378-382.

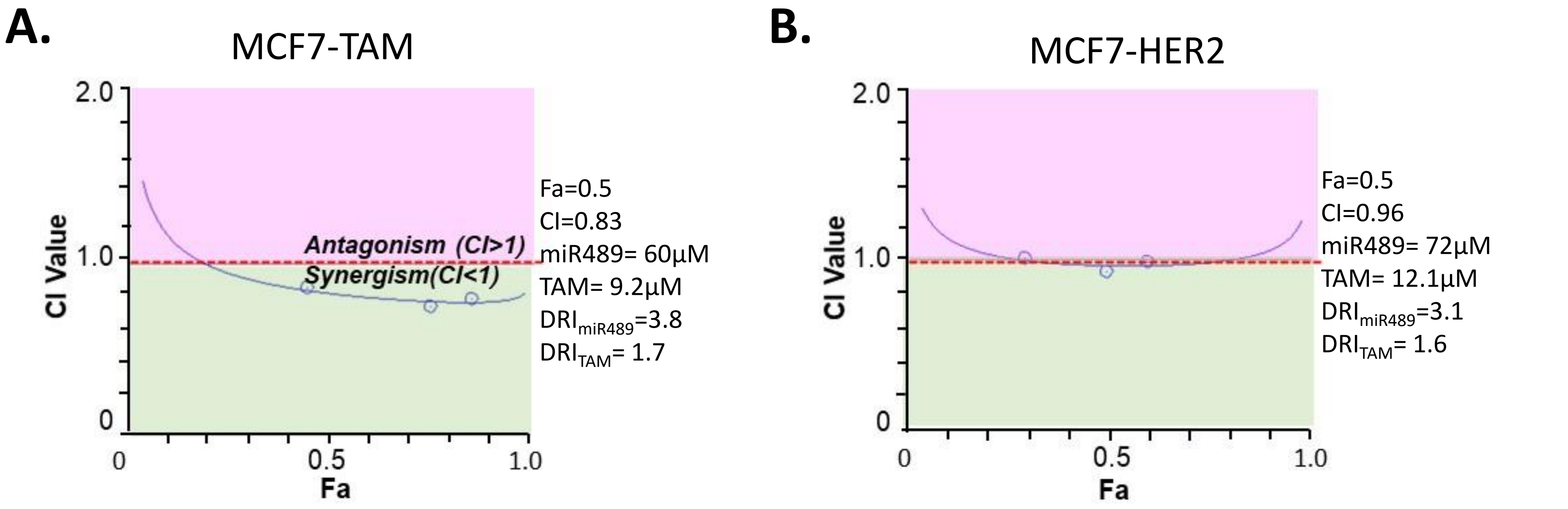

Supplementary Figure S1

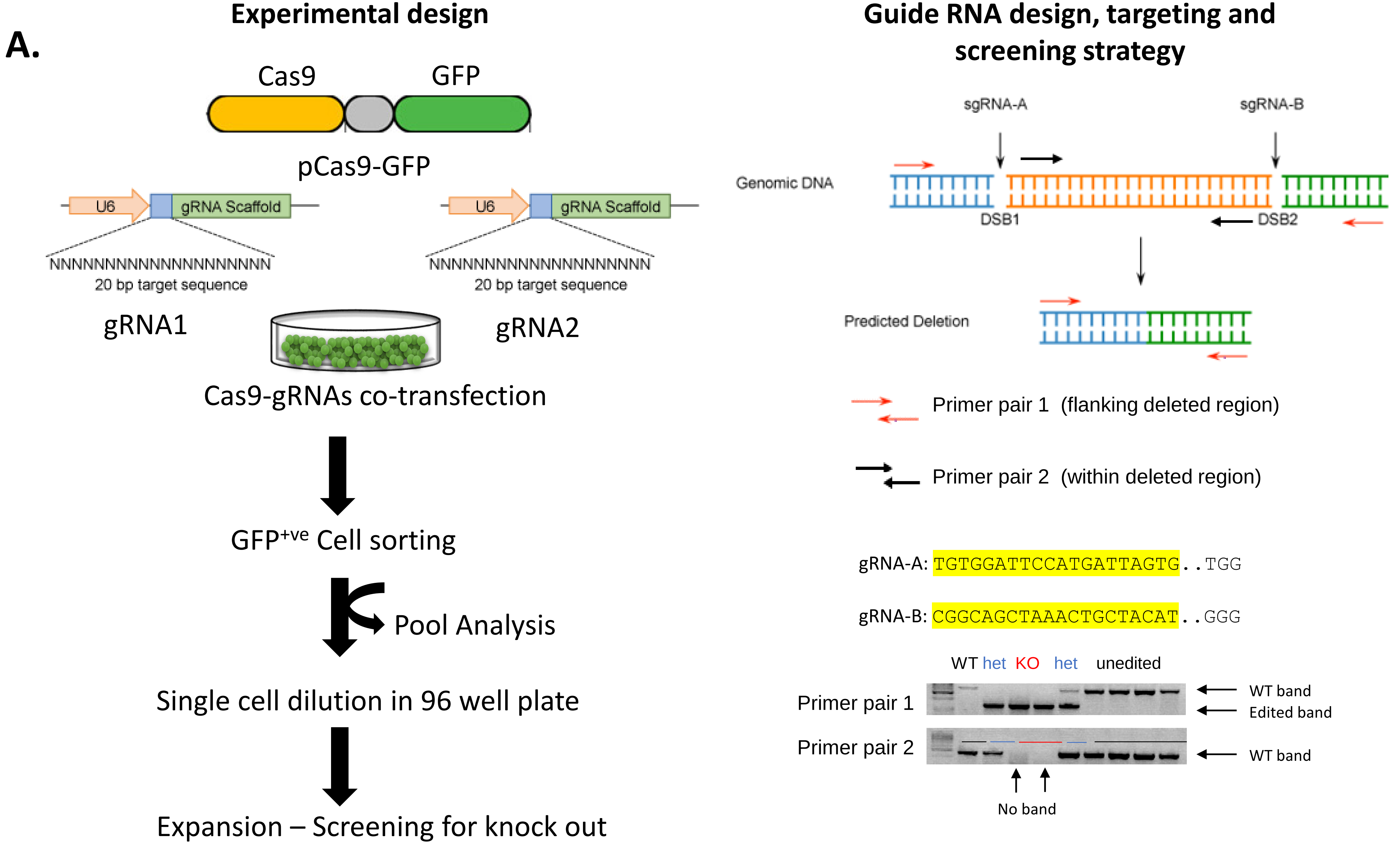

Supplementary Figure S2

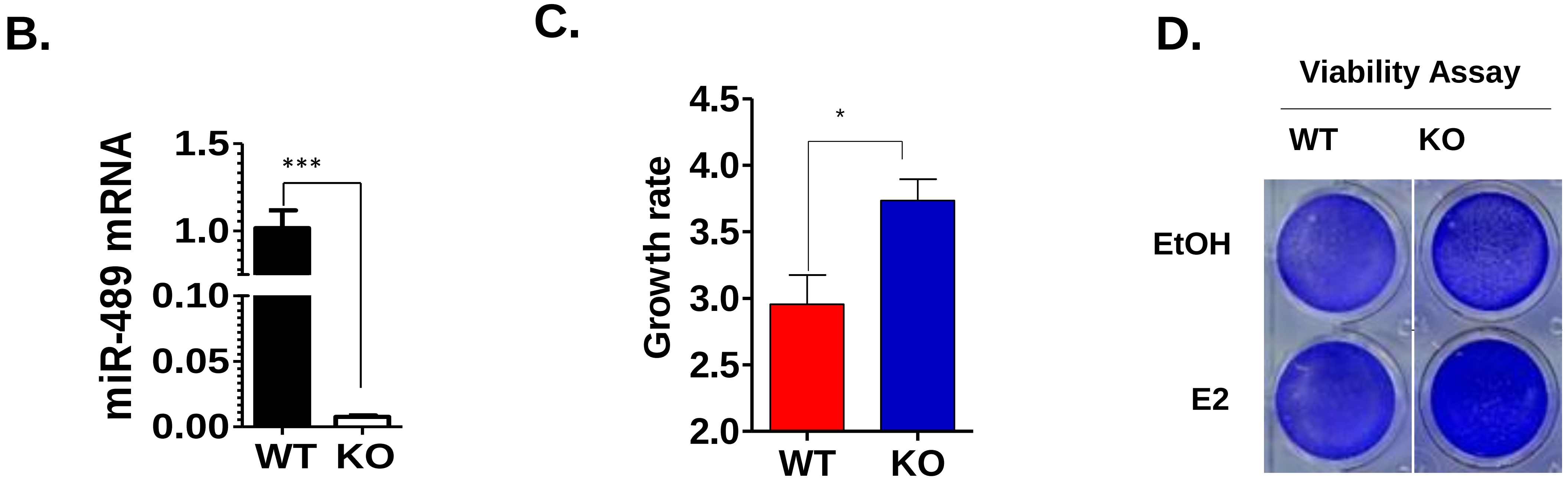

Supplementary Figure S2

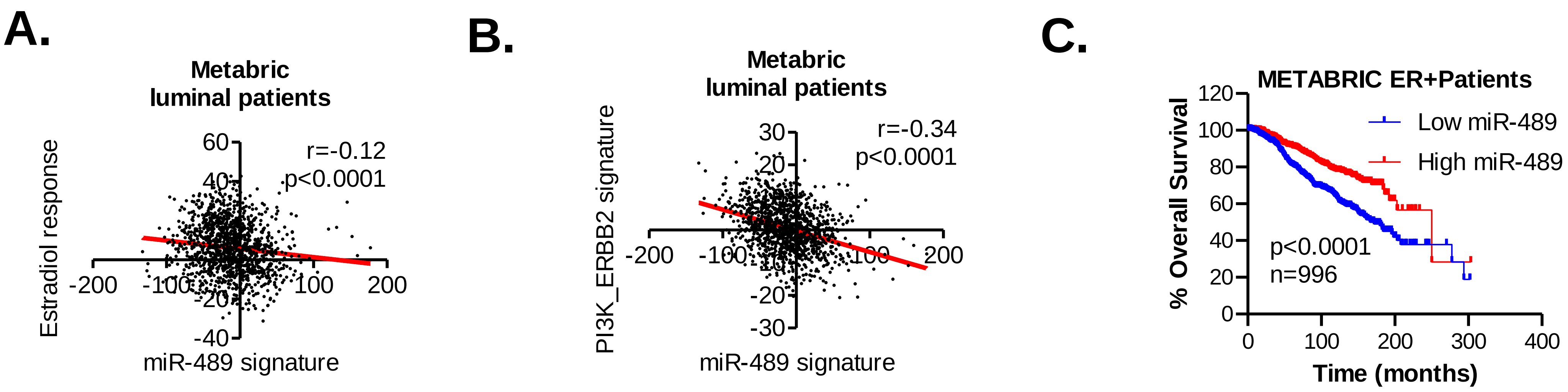

Supplementary Figure S3

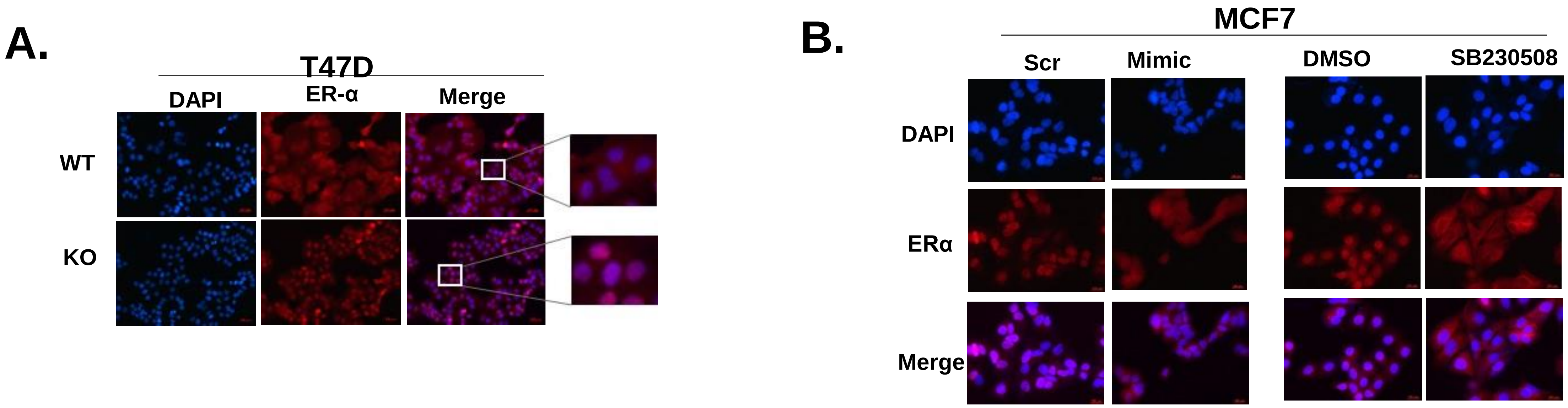

Supplementary Figure S4

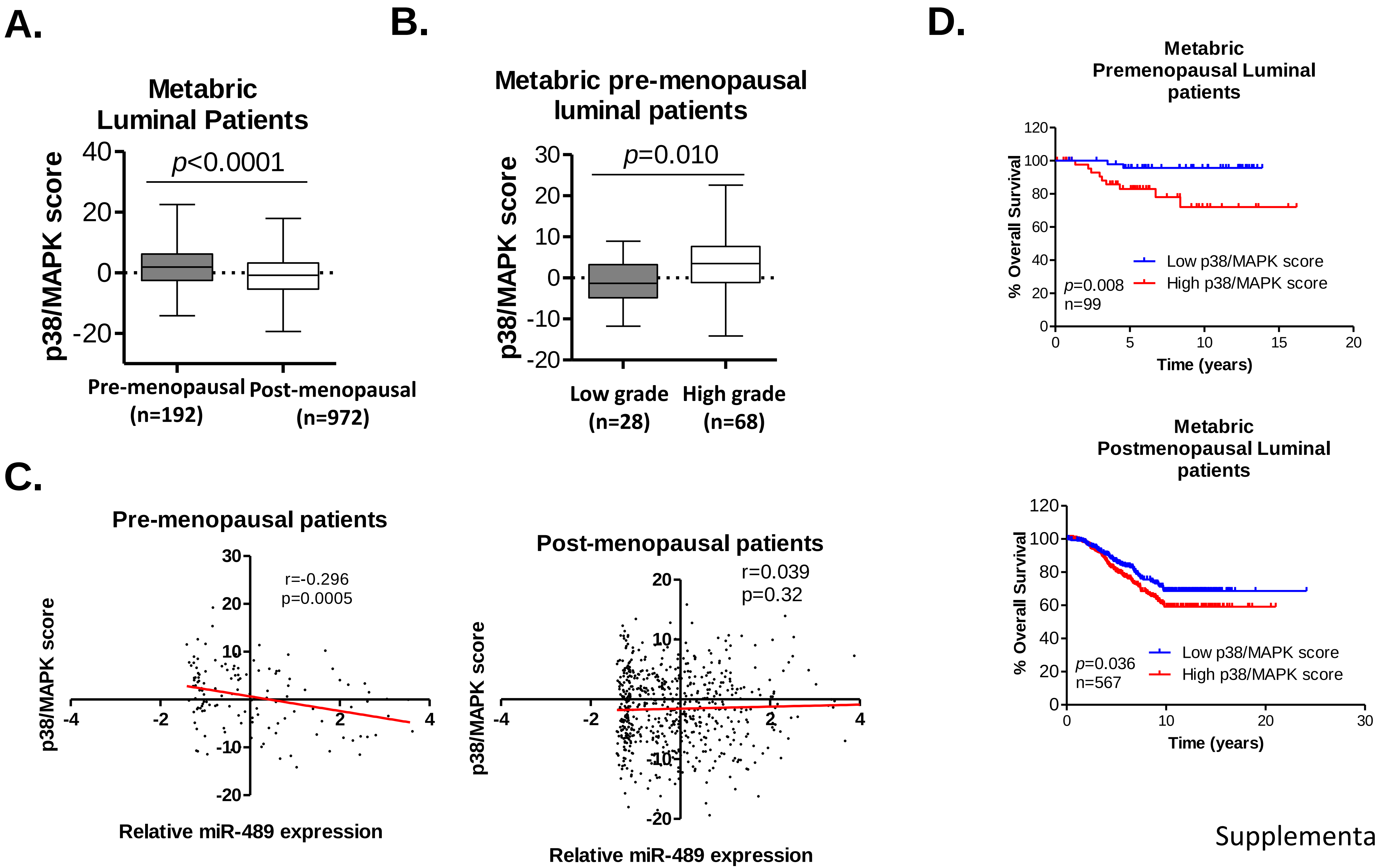

Supplementary Figure S5
